# Sorafenib for the Treatment of Unresectable Hepatocellular Carcinoma: Preliminary Toxicity and Activity Data in Dogs

**DOI:** 10.3390/cancers12051272

**Published:** 2020-05-18

**Authors:** Laura Marconato, Silvia Sabattini, Giorgia Marisi, Federica Rossi, Vito Ferdinando Leone, Andrea Casadei-Gardini

**Affiliations:** 1 Department of Veterinary Medical Sciences, University of Bologna, via Tolara di Sopra 50, Ozzano dell’Emilia, 40064 Bologna, Italy; silvia.sabattini@unibo.it; 2 Biosciences Laboratory, Istituto Scientifico Romagnolo per Lo Studio e La Cura Dei Tumori (IRST) IRCCS, Via Piero Maroncelli 40, Meldola, 47014 Forlì-Cesena, Italy; giorgia.marisi@irst.emr.it; 3 Centro Oncologico Veterinario, via San Lorenzo 1-4, Sasso Marconi, 40037 Bologna, Italy; rossi@centroncologicovet.it (F.R.); leone@centroncologicovet.it (V.F.L.); 4 Division of Oncology, Department of Oncology and Hematology, University Hospital Modena, 41124 Modena, Italy; casadeigardini@gmail.com

**Keywords:** dog, hepatocellular carcinoma, metronomic therapy, outcome, sorafenib, toxicity, spontaneous model

## Abstract

Unresectable nodular and diffuse hepatocellular carcinoma (HCC) have a poor prognosis with limited treatment options. Systemic traditional chemotherapy has been only rarely reported, with unsatisfactory results. The aim of this prospective, non-randomized, non-blinded, single center clinical trial was to investigate safety profile, objective response rate, time to progression and overall survival of sorafenib in comparison with metronomic chemotherapy (MC) consisting of thalidomide, piroxicam and cyclophosphamide in dogs with advanced, unresectable HCC. Between December 2011 and June 2017, 13 dogs were enrolled: seven received sorafenib, and six were treated with MC. Median time to progression was 363 days (95% CI, 191–535) in dogs treated with sorafenib versus 27 days (95% CI, 0–68) in dogs treated with MC (*p* = 0.044). Median overall survival was 361 days (95% CI, 0–909) in dogs receiving sorafenib, while 32 days (95% CI, 0–235) in those receiving MC (*p* = 0.079). Sorafenib seems to be a good candidate for the treatment of dogs with advanced HCC, due to a benefit in disease control and an acceptable safety profile, offering a good basis on which new randomized prospective clinical trials should be undertaken to compare the efficacy and drawback of sorafenib versus MC or traditional chemotherapy.

## 1. Introduction

In dogs, hepatocellular carcinoma (HCC) is the most common primary liver tumor [1,2], and its prognosis depends on the morphological type. The massive form is typically confined to one liver lobe and may be amenable to surgical resection [3]. Also, the metastatic potential is generally low to moderate. Conversely, the nodular and diffuse forms of HCC typically involve multiple liver lobes and are not amenable to surgical resection [1,2,3]. Also, the metastatic rate exceeds 90% for both forms [1,2,3]. Thus, unresectable nodular or diffuse HCC harbors a poor prognosis, as there are limited treatment options for non-surgical cases.

Interdisciplinary approaches, including local tumor ablation, transarterial embolization, and radiotherapy, remain uninvestigated in veterinary medicine. Systemic traditional chemotherapy has only been rarely reported, with unsatisfactory results. Resistance of tumor cells to chemotherapeutic drugs is an important component of clinical treatment failure and recurrence for patients with HCC [4]. In one study, seven dogs with unresectable HCC were treated with intravenous gemcitabine [5]. Median progression-free interval and median survival time were 150 days and 197 days, respectively [5]. In another study, two dogs with unresectable and metastatic HCC did not respond to gemcitabine and carboplatin [6]. Thus, new treatment approaches are urgently needed.

Metronomic chemotherapy (MC) has gained traction recently as an attractive treatment modality due to its favorable toxicity profile and ease of administration in comparison to traditional chemotherapy. MC refers to the practice of administering cytotoxic drugs without prolonged drug-free breaks and at doses significantly lower than dose-intense chemotherapy, with the therapeutic target of both anti-angiogenic and immune-modulatory effects [7]. In veterinary oncology, MC is mainly used in a palliative setting. Drugs that are commonly used in metronomic regimens include, among others, cyclophosphamide and piroxicam [8,9,10]. There are fewer reports of thalidomide use in dogs, either used as a single agent or as part of a metronomic regimen in combination with cyclophosphamide and piroxicam. These reports have shown a favorable toxicity profile and some antitumor activity [11,12,13,14,15].

In people with advanced or intermediate stage HCC, in those with refractory cancer, or in patients no longer amenable to locoregional therapies and preserved liver function (child pugh A), the multi-kinase inhibitor sorafenib remains the only FDA-approved first-line treatment option for systemic therapy [16]. Sorafenib is a multi-kinase inhibitor that targets Raf kinase, vascular endothelial growth factor receptors 1, 2, and 3, and platelet-derived growth factor receptor β with anti-proliferative and anti-angiogenic activities. Unfortunately, in a substantial percentage of human patients, acquired sorafenib resistance remains a major clinical obstacle, leading to treatment failure. Several mechanisms are implicated in the reduction of tumor cell sensitivity to sorafenib, including metabolic rewiring [17,18,19,20,21,22].

The most common drug-related side effects are fatigue, hypertension, anorexia, diarrhea, rash/desquamation, and hand–foot skin reaction [16,23].

In dogs, the drug sorafenib showed potent antitumor activity against canine osteosarcoma and hemangiosarcoma cells in vitro [24,25]. According to preclinical toxicology studies conducted in healthy female beagle dogs, sorafenib administered orally at dosages of 30 mg/kg/day was associated with significant gastrointestinal, cutaneous, renal, adrenal, bone and hematologic toxicity after long term administration (3–12 months), whereas at a 10 mg/kg/day dosing there were no significant side effects observed [26,27]. To date sorafenib was also evaluated clinically in an early phase tolerability study on dogs with various cancers, including one animal with HCC [28]. Single oral doses of sorafenib were tolerable up to 3 mg/kg when given on a once-weekly basis [28]. Thus, it seems plausible that the dose can be further escalated.

The aim of this prospective, non-randomized, non-blinded, single center clinical trial was to investigate safety profile, objective response rate (RR), time to progression (TTP) and overall survival (OS) of sorafenib in comparison with MC consisting of thalidomide, piroxicam and cyclophosphamide in dogs with advanced, unresectable HCC. It was hypothesized that sorafenib would lead to a better outcome than MC.

## 2. Results

### 2.1. Dogs and Tumor Characteristics

Between December 2011 and June 2017, 13 client-owned dogs were enrolled at one single Centre (Centro Oncologico Veterinario): seven received sorafenib, and six were treated with MC.

Represented breeds are listed in Table 1. There were 10 females (seven spayed) and three intact males. Median age was 11 years (range, 5 to 13 years), and median weight was 10.6 kg (range, 2.7 to 41.1 kg).

Complete staging diagnostic tests were performed in each case on the day of initial presentation: nine dogs underwent TBCT, whereas four dogs were staged by means of abdominal ultrasound and thoracic radiographs.

Imaging workup revealed a hepatic round mass with a mean maximum diameter of 6.4 cm (range, 3 to 10 cm). For dogs undergoing TBCT, HCC had a heterogeneous enhancement; whereas for those undergoing abdominal ultrasound, the tumor appeared sonographically heterogeneous solid.

In nine dogs, HCCs appeared as a single large mass located in the quadrate lobe (*n* = 3), the left lateral and medial (*n* = 4), the right lateral (*n* = 1) and right medial lobe (*n* = 1). In three of these nine cases, secondary nodules were observed adjacent to the primary lesion, compatible with intrahepatic metastases.

In the other four cases, multiple hepatic nodules of similar size disseminated in all liver lobes were found, ranging from 0.4 and 9 cm in diameter. Additional findings were regional lymph node enlargement (*n* = 5), peritoneal nodules and ascites (*n* = 2), sternal lymphadenopathy (*n* = 1), thoracic nodules (*n* = 1) and a 2 cm right adrenal nodule (*n* = 1).

**Figure 1:**
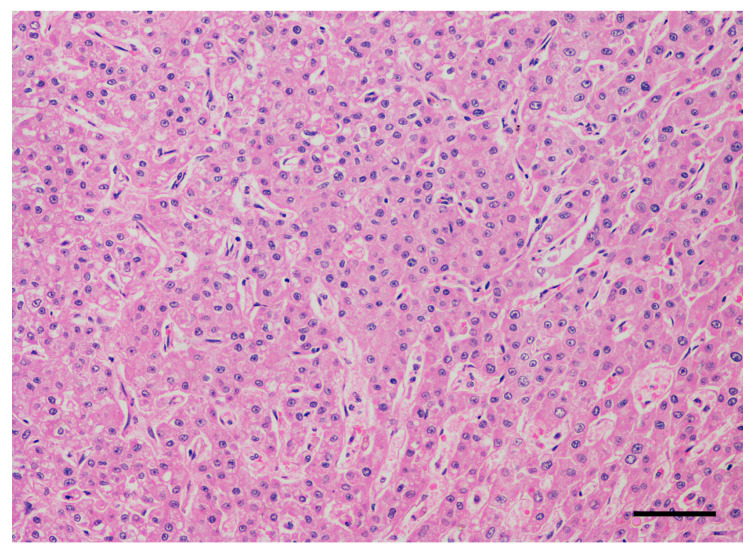
Dog, liver. Histological sample of hepatocellular carcinoma. The tumor is well-differentiated and composed by variably thick trabeculae formed by neoplastic hepatocytes (trabecular pattern). Hematoxylin and eosin. Bar, 100 µm.

**Figure 2:**
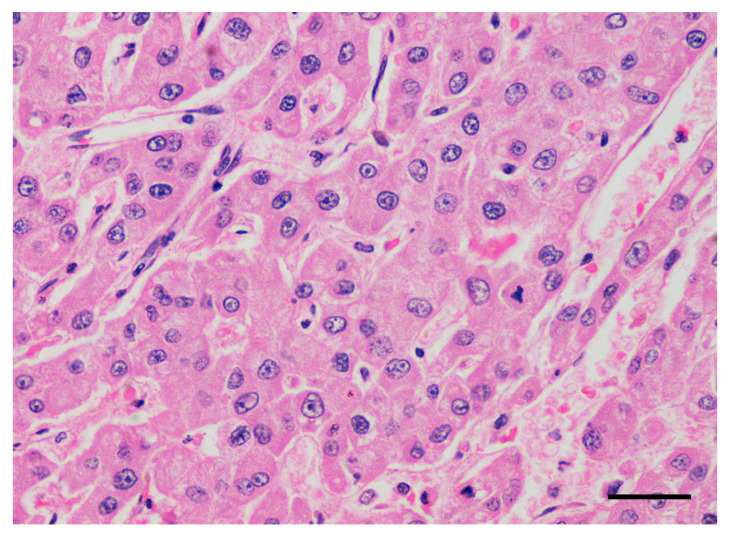
Dog, liver. Histological sample of hepatocellular carcinoma. At high magnification, neoplastic cells show moderate anisocytosis and anisokaryosis, prominent nucleoli and mitotic figures. Hematoxylin and eosin. Bar, 50 µm.

**Figure 3:**
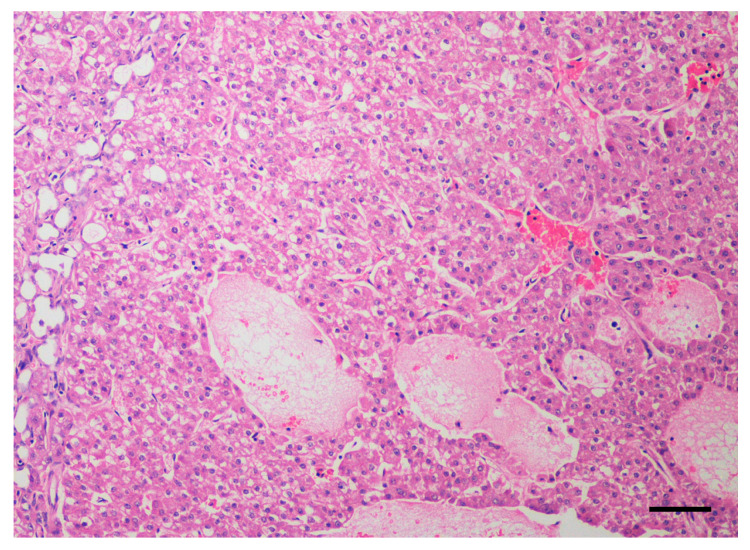
Dog, liver. Histological sample of hepatocellular carcinoma. The trabeculae of hepatocytes are separated by multiple irregular vascular channels and cavernous spaces. The cytoplasm of neoplastic cell is often pale staining or vacuolated due to glycogen or lipid filling. Multifocally, the neoplastic tissue is loss and replaced by granular eosinophilic and karyorrhectic debris (colliquative necrosis). Hematoxylin and eosin. Bar, 100 µm.

A histopathological diagnosis of HCC was obtained in all dogs by means of imaging-guided tru-cut biopsy (*n* = 10) or surgical biopsy (*n* = 3). Histologically, all tumors were characterized by a predominant trabecular pattern, with neoplastic cells arranged in irregular trabeculae up to 20 cells thick often separated by dilated sinusoids, occasionally forming cavernous spaces (Figure 1 and Figure 2). Colliquative necrosis was a common finding (Figure 3). Less frequently, areas of pseudoglandular or solid differentiation were observed in a subset of tumors.

At enrollment, all dogs had measurable HCC: 3 dogs had T2N0M1 disease (stage IV), 3 dogs had T3N0M0 disease (stage III), 2 had T2N1M0 disease (stage IV), 2 had T3N1M1 disease (stage IV), 2 dogs had T3N1M0 disease (stage IV), and one dog had T1N2M0 disease (stage IV). Enlarged regional lymph nodes and other suspected metastatic lesions were sampled by ultrasound-guided fine-needle aspiration in all N1 or M1 cases, confirming metastatic disease.

Five dogs had a normal CBC, renal and hepatic function, as documented by pre-therapeutic bloodwork. Eight dogs had an increased ALP (4–53 times the upper reference limit), three dogs had an increased GGT (2.2–569 times the upper reference limit), two dogs had an increased bilirubin (2 and 3 times the upper reference limit, respectively), and one dog had an increased AST (2.5 times the upper reference limit).

Of the seven dogs receiving sorafenib, two underwent prior surgical debulking of the largest hepatic lesion, 38 and 42 days prior to enrollment, respectively. Both dogs had incomplete margins based on histopathological evaluation and measurable regional metastatic lymph nodes at enrollment. These dogs had the longest TTP (1250 and 1579 days, respectively) and were both still alive at data analysis closure, after 1398 and 1706 days.

Of the six dogs receiving MC, one underwent prior surgical debulking of the largest hepatic lesion 31 days before initiation of MC; two hepatic lesions and metastasis to the regional lymph nodes and lungs were present at enrollment. This dog survived 27 days only after initiation of MC.

Demographic features and potential prognostic variables were homogeneously distributed between the two treatment groups (Table 2).

### 2.2. Treatment and Toxicity

Seven dogs received oral sorafenib. Median treatment duration was 228 days (range, 168 to 321 days). At data analysis closure, five dogs had discontinued treatment due to drug unavailability, and one dog due to PD after 168 days. One dog had died for tumor-unrelated causes (leishmaniasis) while still under treatment. The dog experiencing PD received one trans-arterial chemoembolization after having interrupted sorafenib. Two of the five dogs that discontinued treatment due to drug unavailability were crossed over to MC, whereas the remaining three received no further treatment based on owners’ preference. Interestingly, two of the latter were still alive in CR and PR, respectively, at data analysis closure, whereas the other 2 switching to MC progressed and died due to tumor-related causes after 28 and 34 days from crossover.

Six dogs were treated with MC. Median treatment duration was 21 days (range, 4 to 703 days). Only one dog interrupted treatment due to PD after 703 days and was treated with toceranib in a rescue setting.

Concerning toxicity, in the sorafenib group three dogs developed cutaneous toxicity, consisting of one each of the following: grade 2 alopecia, grade 1 hyperpigmentation, and grade 1 localized erythema and alopecia. One dog also developed grade 1 diarrhea that resolved uneventfully with symptomatic treatment. Routine liver tests were monitored during sorafenib treatment. Hyperbilirubinemia and serum transaminase elevations did not occur.

In the MC group, three dogs experienced gastrointestinal toxicity (2 of grade 1, 1 of grade 2), one dog experienced grade 1 hemorrhagic cystitis, and one dog experienced grade 1 renal toxicity.

In the two groups, treatment-related mortality was not observed.

Based on the questionnaire results, QoL was improved in 6 of the 7 dogs receiving sorafenib, whereas in one dog QoL was maintained. Only 3 of the 6 dogs receiving MC lived long enough to assess QoL. In two of them, QoL was improved, whereas in the last dog QoL was maintained.

### 2.3. Response Rate and Outcome

Of the seven dogs treated with sorafenib, 1, 3, and 3 dogs had CR, PR, and SD, respectively. CR was documented in one dog that underwent hepatic debulking and where hepatic, splenic, and sternal lymphadenopathy was evident at presentation, resolving at the first imaging follow-up and until 1706 days. PR was observed in three dogs and consisted of a decrease of the liver lesions size (*n* = 3), resolution of malignant effusion (*n* = 2) and reduction of lung nodules (*n* = 1). The response rate was 57.1%, and the disease-control rate was 100%.

Of the six dogs receiving MC, three dogs had SD and three dogs had PD. Among the three dogs with SD, the hepatic mass size was stable respectively for 35, 219 and 703 days and subsequently increased in size with lesion rupture and secondary haemoabdomen. The response rate was 0% and the disease-control rate was 50%.

At the end of the study, five dogs receiving sorafenib had died (4 due to PD and 1 due to leishmaniasis). Tumor-related deaths were due to rupture of HCC with hemoabdomen (*n* = 2) and convulsive crisis (*n* = 2), most likely attributable to hepatic encephalopathy. Because necropsy was not permitted, cerebral metastases could not be completely ruled out in these cases. Median TTP was 363 days (95% CI, 191–535); median OS was 361 days (95% CI, 0–909).

All dogs in the MC group had died due to PD. Tumor-related deaths were due to rupture of HCC with hemoabdomen (*n* = 4), peritoneal and adrenal metastases (*n* = 1), and disseminated intravascular coagulopathy (*n* = 1). Median TTP was 27 days (95% CI, 0–68); median OS was 32 days (95% CI, 0–235).

**Figure 4:**
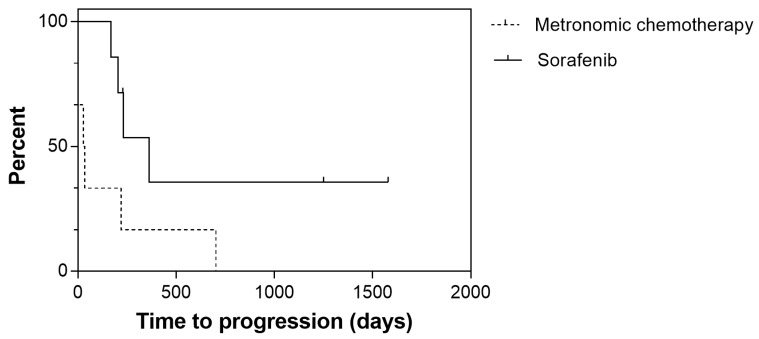
Time to progression for 13 dogs with hepatocellular carcinoma treated with sorafenib (solid line) or metronomic chemotherapy (dashed line). In the sorafenib group, dogs had a longer time to progression (363 versus 27 days; *p* = 0.044).

**Figure 5:**
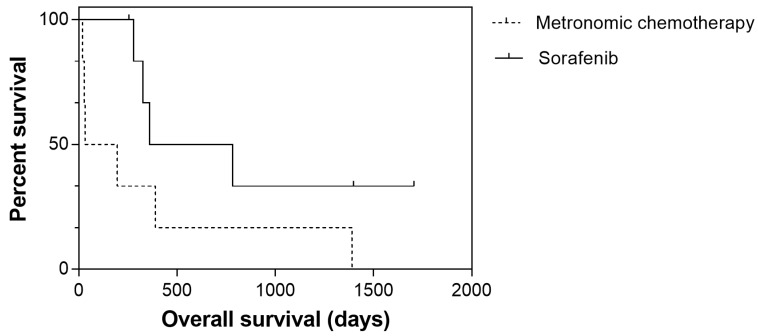
Overall survival for 13 dogs with hepatocellular carcinoma treated with sorafenib (solid line) or metronomic chemotherapy (dashed line). Median overall survival was 361 days for the sorafenib group and 32 days for the metronomic chemotherapy group; however, the difference was not statistically significant (*p* = 0.079).

TTP was significantly longer among dogs receiving sorafenib (*p* = 0.044), whereas no significant difference was observed for OS (*p* = 0.079) (Figure 4 and Figure 5).

None of the other examined variables were significantly associated with TTP or OS (Table 3).

## 3. Discussion

The aim of this study was to evaluate the safety and efficacy of sorafenib in dogs with unresectable HCC compared with those that received MC.

MC is based on the principle of administering low doses of chemotherapeutic drug continuatively for long periods of time, resulting in an increased antitumor activity through the inhibition of neo-angiogenesis and at the same time in a low toxicity profile due to the reduced dose of drug at each administration. On the wave of the good results reported by our center in treating dogs with various advanced cancer [11,14,15], we tested MC in canine HCC, confirming a favorable safety profile. In people with HCC, MC with capecitabine showed a good safety profile and antitumor efficacy [29,30].

While the toxicity profile of MC is well documented, little is known about sorafenib. When the current clinical trial was started, there were no data concerning treatment toxicity in dogs, thus the dose that was considered to be safe in healthy beagles was adopted [26,27].

The schedule and dosage of sorafenib in the treatment of HCC used in the present study was found to be safe and tolerable. Three (42.9%) cases of cutaneous toxicity and 1 (14.3%) case of diarrhea occurred as adverse events, but none of them were dose-limiting toxicities. However, within the realm of this small non dose-escalating trial, our results need confirmation by other studies. Notably, the analysis of QoL revealed an improvement in 6/7 dogs receiving sorafenib.

Conversely, sorafenib has significant toxicity in humans, and the most common treatment-related adverse events include hand-foot syndrome, hypertension, and diarrhea [16]. Nevertheless, according to several studies, the development of one or more adverse events was associated with longer TTP, disease control rate and OS [16].

It was reported that in dogs with unresectable HCC there are no efficacious non-surgical treatment options [1,2,3]. Currently, there is no standard systemic chemotherapy with cytotoxic agents for dogs with diffuse or nodular HCC. Traditional chemotherapy with gemcitabine as single agent or alternated to carboplatin was tested in dogs, with no evidence of survival benefit [5,6].

Sorafenib is the first molecular targeted drug approved for the treatment of human advanced HCC and is a potent small molecule inhibitor of multiple kinases with anti-proliferative and anti-angiogenic activities.

After 10 years of research into sorafenib in human advanced HCC, there are still no validated prognostic or predictive factors of response. Moreover, the exact molecular mechanisms of resistance have not been fully elucidated [31,32].

Our population treated with sorafenib had an improved TTP in comparison with the dogs treated with MC. Similarly, although not formally compared, the disease response rate, TTP and OS observed in this study were higher than the outcomes reported by other studies focusing on cytotoxic chemotherapy [5,6].

The small sample size did not allow determining if this observation translated into a better survival outcome, although dogs receiving sorafenib showed a median OS (361 days) 10 times higher than those receiving MC (32 days). It must be noted that five out of the seven dogs receiving sorafenib discontinued treatment due to drug unavailability. Thus, it may be possible that continuous therapy with sorafenib would have translated into a prolonged survival as well.

On the other hand, although unlikely, the trend to live longer in the sorafenib group may be due to the lack of effective treatments for dogs with unresectable HCC that failed MC. Indeed, dogs receiving sorafenib were allowed to crossover to MC or other treatments if they progressed or if the drug was no longer available, whereas no post-progression therapy was recommended for those dogs on MC. Also, owners willing to treat their dogs with an investigational drug may be willing to continue treatment once the disease progresses, especially in the face of an improved QoL. This may have led to a better outcome in the group of dogs receiving sorafenib. It must be acknowledged that in veterinary oncology, quality (rather than quantity) of life is the primary goal of any antitumoral treatment. As a consequence, survival time is intimately linked to QoL, possibly biasing outcome results. However, it is important to note that dogs crossing over received MC for a short time (28 and 34 days, respectively), thereby reducing the likelihood of a significant survival benefit attributable to the therapeutic switch.

In the current study, two dogs in the sorafenib treatment arm were pretreated with debulking surgery prior to the beginning of systemic therapy and were exceptional responders despite the presence of residual local and metastatic disease. This finding was not unexpected, as bulky disease is challenging to treat with current systemic therapies for non-hematologic cancers. According to the Norton-Simon hypothesis, the highest degree of chemosensitivity occurs when the tumor is not bulky. As such, a debulking surgery may result in a residual tumor burden that is more chemosensitive, implying that the ability to receive local therapy could have contributed to the better outcome in these dogs.

Beside the small population size, the present study has additional limitations. First, there was no randomization, and the treatments were selected at the clinician and owners’ discretion. This may have resulted in a selection bias for dogs treated with sorafenib, although there were no significant differences in the dogs’ characteristics between the two groups Second, some dogs in both groups received prior debulking surgery. Lastly, low sorafenib unavailability precluded continued use of sorafenib in five out of seven dogs.

With these limitations in mind, sorafenib seems to be a good candidate for the treatment of dogs with advanced HCC, due to a benefit in disease control and an acceptable safety profile.

## 4. Material and Methods

### 4.1. Inclusion and Exclusion Criteria

Dogs with histologically confirmed HCC of any clinical stage were qualified for recruitment (Table 4). Dogs were eligible for enrollment if they were ineligible for surgical excision or if nodal or distant metastatic disease was confirmed at admission, regardless of prior surgical debulking of the primary tumor.

Inclusion criteria were as follows: (1) at least one unidimensional lesion measurable by ultrasound or computed tomography (CT) according to the canine Response Evaluation Criteria In Solid Tumors version 1.0 (cRECIST v1.0; 23); (2) not being a candidate for surgical resection if clinical stage evaluations indicated stage T3 or N1 or M1, or if imaging revealed insufficient apparently normal hepatic tissue for liver lobectomy to be safely performed; (3) no prior systemic antitumoral treatments; (4) an adequate bone marrow, cardiac, renal, and hepatic function, as documented by a normal CBC, renal and hepatic serum chemistry values. Specifically, dogs were required to have an absolute neutrophil count ≥ 1500 cells/µL, hematocrit ≥ 25%, platelet count ≥ 100,000/µL, serum creatinine concentration and alanine transaminase activity ≤ 3 times the upper limit of normal, lethargy/fatigue status (VCOG-CTCAE version 1.0) of either 0 or 1 [24].

Exclusion criteria included hepatic encephalopathy, second malignancies, concurrent serious systemic diseases, or previous systemic chemotherapy or molecular target therapy.

Enrollment began in December 2011, and the study closed in June 2017.

Written, informed consent to participate in this study was obtained from each owner. All owners were informed of the advantages and disadvantages of the treatment options, including unknown treatment outcomes and treatment-related morbidities. The final treatment decision was made jointly by each owner and attending clinician, with full respect for the option to decline participation.

### 4.2. Treatment Protocol

Sorafenib treatment was offered to all dogs. If owners rejected sorafenib, dogs received MC.

Sorafenib was administered orally at a dose of 5 mg/kg twice daily on an empty stomach; the dose was administered to the nearest 50 mg.

The drug was discontinued in the event of disease progression, protocol-defined unacceptable toxicity, a dose interruption of more than 30 days, owner choice, the recommendation of the attending clinician, or drug unavailability. In case of sorafenib discontinuation, regardless of the reason, dogs were allowed to crossover to alternative treatments, including MC.

MC was administered orally and consisted of low-dose cyclophosphamide (10 mg/m2 q24 h), piroxicam (0.3 mg/kg q24 h) and thalidomide (2 mg/kg q24 h). Owners administering thalidomide were comprehensively informed of its known teratogenic effect.

### 4.3. Safety Monitoring and Efficacy of Treatment

Safety evaluation was performed 14 days after the beginning of treatment, and included medical history obtained from the owners, physical examination, CBC with differential and platelet count, serum biochemical analysis, and urinalysis with microscopic examination of urine sediment. The dog’s vital signs, temperature and systolic blood pressure were recorded at each visit.

During follow-up, the levels of ALT, AST, γGTP, serum albumin, total bilirubin, urea, and creatinine were determined every 4–6 weeks to evaluate liver function. Toxic effects were graded in accordance with VCOG-CTCAE guidelines [33]. Unacceptable toxicity was defined as side effects of grade 3 or more in any tissue or organ.

Quality of life (QOL) was assessed using a questionnaire designed for the study based on investigators’ clinical expertise and this was assessed at baseline (before starting treatment) and at every recheck during treatment (Questionnaire S1).

Tumor measurements, based on RECIST [34], were performed at baseline (within 14 days before initiation of treatment) by means of abdominal ultrasound or total body computed tomography (TBCT). Thoracic radiographs were also included in the initial work-up and repeated in the follow-up if clinically indicated. The first follow-up imaging was done after 4 weeks in both treatment arms and every 4–8 weeks thereafter.

The therapeutic effect was defined by the RECIST as follows: complete response (CR), disappearance of all measurable lesions for >4 weeks; partial response (PR), >30% decrease in the sum of the largest target lesion diameters and no development of a new lesion for >4 weeks; progressive disease (PD), >20% increase in the sum of the largest target lesion diameters or appearance of a new lesion; and stable disease (SD), neither PR nor PD seen for >4 weeks.

Dogs that died before their first radiographic assessment were classified as having PD. The number of dogs achieving CR, PR, SD, and PD was analyzed. The response rate was defined as the percentage of dogs whose best response RECIST rating of CR or PR was maintained for at least four weeks after the first demonstration of such a rating. The disease control rate was defined as the percentage of dogs whose best response RECIST rating of CR, PR, or SD was maintained for at least four weeks after the first demonstration of such a rating. For dogs with SD or an objective response, follow-up assessments for survival were performed every 4 weeks until PD and/or death.

### 4.4. Statistical Analysis

Differences in the demographic and clinical features between dogs receiving the two protocols were evaluated with Mann–Whitney U-test and Fisher’s exact test.

Time to progression (TTP) was defined as the time from the initiation of treatment to the date of disease progression. Overall survival (OS) was defined as the time from the initiation of treatment to the date of death or the dog’s last follow-up. For survival analysis, dogs were censored if they were alive at the time of study closure or died of tumor-unrelated causes, whereas for TTP dogs were censored if, by the last examination, disease had not progressed.

Survival curves were generated according to the Kaplan-Meier product-limit method. Survival estimates are presented as medians with the corresponding 95% confidence intervals (95% CI). The influence of potential prognostic variables (sex, age, weight, multiplicity of lesions, clinical stage, altered liver enzymes, surgical debulking, treatment protocol, treatment toxicity) on TTP and OS was investigated with the log-rank test.

Data were analyzed by use of commercial software programs (SPSS Statistics v. 24, IBM, Somers, NY, and Prism v. 5.0, GraphPad, San Diego, CA, USA). *p* values ≤ 0.05 were considered significant.

## 5. Conclusions

The results of this study offer a good basis on which new randomized prospective clinical trials should be undertaken to compare the efficacy and drawback of sorafenib versus MC or traditional chemotherapy in dogs with advanced HCC.

## Figures and Tables

**Table 1:** Demographic and clinical details of 13 dogs with hepatocellular carcinoma.

Breed	Sex	Age	Weight	Clinical Stage	Altered Liver Enzymes	Surgical Debulking	Treatment Protocol	Treatment Toxicity	Antitumor Response, TTP	OS (Cause of Death)
Golden retriever	M	11	41.1	T2N0M1	Yes	No	Sorafenib	G1 erythema and alopecia	PR, 363	784 (cancer)
Golden retriever	SF	5	27.8	T1N2M0	No	Yes	Sorafenib	G1 hyperpigmentation; G1 diarrhea	CR, 1579 (no progression)	1706 (alive)
Yorkshire terrier	SF	10	3.6	T2N1M0	Yes	Yes	Sorafenib	G2 alopecia	PR, 1250 (no progression)	1398 (alive)
Bloodhound	F	8	20.5	T3N1M1	No	No	Sorafenib	None	PR, 228 (no progression)	255 (leishmaniasis)
Cross-breed	M	11	10	T3N0M0	Yes	No	Sorafenib	None	SD, 168	361 (cancer)
Pomeranian	SF	13	10.1	T2N1M0	Yes	No	Sorafenib, Then MC crossover	None	SD, 230	327 (cancer)
Poodle	F	13	2.7	T2N0M1	No	No	Sorafenib, Then MC crossover	None	SD, 205	279 (cancer)
Shih-tzu	SF	11	6.7	T3N1M1	Yes	Yes	MC	None	PD, 27	27 (cancer)
Beagle	SF	11	13.1	T3N1M0	Yes	No	MC	None	SD, 703	1390 (cancer)
Scottish terrier	SF	7	10.6	T2N0M1	Yes	No	MC	G1 gastrointestinal; G1 renal	SD, 35	196 (cancer)
French bouledogue	SF	6	11.7	T3N0M0	No	No	MC	G1 hemorrhagic cystitis	SD, 219	390 (cancer)
Jack Russell Terrier	M	12	8.1	T3N0M0	Yes	No	MC	None	PD, 1	19 (cancer)
German Shepherd	F	11	29.6	T3N1M0	No	No	MC	G2 gastrointestinal	PD, 1	32 (cancer)

M = male; F = female; SF = spayed female; MC = metronomic chemotherapy; G = grade; TTP = time to progression; OS = overall survival; CR = complete remission; PR = partial remission; SD = stable disease; PD = progressive disease.

**Table 2:** Demographic information and distributions of variables potentially associated with prognosis of 13 dogs with hepatocellular carcinoma treated with sorafenib or metronomic chemotherapy.

Variable	Sorafenib (*n* = 7)	Metronomic Chemotherapy (*n* = 6)	*p*
Sex			>0.999
Male	2	1	
Female	5	5	
Age ^a^			>0.999
≤11 Years	5	5	
>11 Years	2	1	
Weight ^a^			0.592
≤10.6 kg	4	2	
>10.6 kg	3	4	
Surgical Debulking			>0.999
No	5	5	
Yes	2	1	
Clinical Stage			>0.999
III	2	1	
IV	5	5	
Hepatic Enzymes			>0.999
Within Normal Limits	3	2	
Altered	4	4	
Treatment Toxicity			>0.099
No	4	3	
Yes	3	3	

^a^ Median used as cut-off value.

**Table 3:** Log-rank test for the variables potentially associated with increased risk of tumour progression and overall survival in 13 dogs with hepatocellular carcinoma.

Variable	Time to Progression		Overall Survival	
	Median (95% CI)	*p*	Median (95% CI)	*p*
Sex		0.301		0.474
Male	168 (0–435)		327 (172–481)	
Female	219 (184–253)		361 (0–908)	
Age ^a^		0.235		0.059
≤11 Years	219 (0–488)		390 (0–979)	
>11 Years	205 (0–531)		212 (0–695)	
Weight ^a^		0.237		0.194
≤10.6 kg	168 (0–509)		279 (66–492)	
>10.6 kg	363 (81–645)		784 (0–1606)	
Surgical Debulking		0.107		0.104
No	205 (126–284)		327 (213–441)	
Yes	not reached		not reached	
Clinical Stage		0.102		0.330
III	168 (0–435)		361 (0–908)	
IV	230 (18–442)		327 (0–1030)	
Hepatic Enzymes		0.643		0.675
Within Normal Limits	219 (189–249)		390 (93–687)	
Altered	168 (0–438)		327 (98–556)	
Treatment		0.044 *		0.079
Metronomic Chemotherapy	27 (0–68)		32 (0–235)	
Sorafenib	363 (191–535)		361 (0–909)	
Treatment Toxicity		0.357		0.194
No	205 (110–300)		327 (1–653)	
Yes	219 (0–613)		390 (0–1096)	

^a^ Median used as cut-off value. * Significant.

**Table 4:** TNM staging system for canine hepatocellular carcinoma.

**Primary tumor (T)**
T0: no evidence of primary tumor
T1: solitary tumor of any size involving one lobe
T2: multiple tumors of any size involving multiple lobes
T3: tumor(s) with direct invasion of adjacent organs
**Regional lymph nodes (N)**
N0: no regional lymph nodes metastasis
N1: regional lymph node metastasis
N2: distant lymph node metastasis
**Distant metastasis (M)**
M0: no distant metastasis
M1: distant metastasis

## Supplementary Materials

The following are available online at https://www.mdpi.com/2072-6694/12/5/1272/s1, Questionnaire S1 Questionnaire for evaluating health-related quality-of-life in 13 dogs with advanced hepatocellular carcinoma receiving metronomic chemotherapy or sorafenib.
