# Supplementary material for: Sorafenib for the Treatment of Unresectable Hepatocellular Carcinoma: Preliminary Toxicity and Activity Data in Dogs
Source: Cancers (Basel). 2020 May 18;12(5):1272. doi: 10.3390/cancers12051272 (PMC7281367; doi:10.3390/cancers12051272)
Supplement: Supplementary file 1 [file cancers-12-01272-s001.pdf]

Article

Sorafenib for the Treatment of Unresectable Hepatocellular Carcinoma: Preliminary Toxicity and Activity Data in Dogs

Laura Marconato, Silvia Sabattini, Giorgia Marisi, Federica Rossi, Vito Ferdinando Leone and Andrea Casadei-Gardini

Questionnaire for evaluating health-related quality-of-life in 13 dogs with advanced hepatocellular carcinoma receiving metronomic chemotherapy or sorafenib. Parameters included behavior (questions 1, 4, and 7), activity (questions 2, 6 and 8), appetite (question 5), and pain (question 3). The questionnaire was scored at baseline and during treatment, and differences were statistically assessed.

1. How much attention is your dog giving to the family?

Totally indifferent (3 points)
Decreased attention (2 points)
Attention has not changed (1 point)

2. Is your pet still active?

My pet lays in one place all day long (3 points)
Occasionally (2 points)
My pet moves and plays in a normal way (1 point)

3. How is your dog sleeping?

Very badly/Not sleeping at all (3 points)
Intermittently (2 points)
Normally (1 point)

4. Does your dog keep its hygienic habits (i.e., does your dog clean itself)?

No (3 points)
Less than before (2 points)
Yes (1 point)

5. Does your dog have an appetite?

No (3 points)
Little, it needs to be forced (2 points)
Normal (1 point)

6. Does your dog show any of the following signs: propulsive circling, head pressing, aimless wandering, weakness, ataxia, amaurosis (unexplained blindness), behavior change (e.g., aggression), collapse, seizures?

Yes (3 points)
Occasionally (2 points)
No (1 point)

7. How is your dog's mood?

Totally altered (3 points)
A bit depressed (2 points)
Normal (1 point)

8. Does your dog get tired easily?

Yes, always (3 points)
Frequently (2 points)
No (1 point)

© 2019 by the authors. Licensee MDPI, Basel, Switzerland. This article is an open access article distributed under the terms and conditions of the Creative Commons Attribution (CC BY) license (http://creativecommons.org/licenses/by/4.0/).
